# Clinical Implications of Amyloid-Beta Accumulation in Occipital Lobes in Alzheimer’s Continuum

**DOI:** 10.3390/brainsci11091232

**Published:** 2021-09-18

**Authors:** Jihye Hwang, Chan Mi Kim, Ji Eun Kim, Minyoung Oh, Jungsu S. Oh, Young Wook Yoon, Jae Seung Kim, Jae-Hong Lee, Jee Hoon Roh

**Affiliations:** 1 Department of Neurology, Asan Medical Center, University of Ulsan College of Medicine, Seoul 05505, Korea; jh.hwang0110@gmail.com (J.H.); chanmi.kim88@gmail.com (C.M.K.); kje8405@daum.net (J.E.K.); jhlee@amc.seoul.kr (J.-H.L.); 2 Department of Neurology, Keimyung University Daegu Dongsan Hospital, Daegu 42601, Korea; 3 MGH/MIT/HMS Athinoula A. Martinos Center for Biomedical Imaging, Massachusetts General Hospital, Harvard Medical School, Charlestown, MA 02129, USA; 4 Department of Radiology, Massachusetts General Hospital, Harvard Medical School, Boston, MA 02114, USA; 5 Department of Neurology, Gangneung Asan Hospital, Gangneung 25440, Korea; 6 Department of Nuclear Medicine, Asan Medical Center, University of Ulsan College of Medicine, Seoul 05505, Korea; my@amc.seoul.kr (M.O.); jungsu.oh@gmail.com (J.S.O.); jaeskim@amc.seoul.kr (J.S.K.); 7 Neuroscience Research Institute, Korea University College of Medicine, Seoul 02841, Korea; ywyoon@korea.ac.kr; 8 Department of Physiology, Korea University College of Medicine, Seoul 02841, Korea

**Keywords:** alzheimer’s disease (AD), amyloid-beta (Aβ), occipital lobe, PET

## Abstract

A substantial amount of amyloid-beta (Aβ) accumulates in the occipital cortices; however, it draws less attention. We investigated the clinical implications of Aβ accumulation in the occipital lobes in the Alzheimer’s disease (AD) continuum. [18F]-Florbetaben amyloid PET scans were performed in a total of 121 AD or amnestic mild cognitive impairment (aMCI) patients. Of the 121 patients, 74 Aβ positive patients were divided into occipital Aβ positive (OCC+) and occipital Aβ negative (OCC−) groups based on Aβ accumulation in the bilateral occipital lobes. The OCC+ group (41/74, 55.4%) was younger and had a younger age at onset than the OCC− group. The OCC+ group also had an increased standard uptake value ratio in the occipital lobes and greater cortical thinning in relevant areas. The OCC+ group had a higher global deterioration scale, lower performance for the copy, immediate recall, delayed recall, and recognition in Rey–Osterrieth Complex Figure tests than the OCC- group, although both groups had similar disease durations. AD or aMCI patients in the OCC+ group exhibited features noted in early onset AD with relevant neuropsychological and image findings. Occipital Aβ positivity in amyloid PET scans need to be considered as an underestimated marker of early onset AD continuum.

## 1. Introduction

The aggregation of beta-amyloid (Aβ) peptides and the formation of Aβ plaques are pathological hallmarks of preclinical and clinical Alzheimer’s disease (AD) [1,2,3]. The main brain regions of Aβ plaque accumulation in AD encompass the association cortices, including the prefrontal, orbitofrontal, parietal, temporal, and cingulate cortices, and the precuneus. Intriguingly, many amyloid PET imaging studies have shown a substantial amount of fibrillar forms of Aβ aggregation in the occipital lobes [4,5,6,7], although such findings and their clinical implications have not been appreciated in the literature. This is probably because the pathological accumulation of Aβ in the occipital cortices could have been interpreted as changes in primary cortices such as the primary motor and sensory cortices. Given the non-trivial and potentially underestimated accumulation of Aβ in the occipital lobes and pathological evidence that indicated existence of Aβ plaques in the occipital cortices [8,9], we investigated whether there was a substantial amount of Aβ in the occipital lobes and how it affected clinical features on the Alzheimer’s continuum, which refers to individuals with biomarker designation of either AD or Alzheimer’s pathologic change [2]. We hypothesized that patients with Aβ accumulation in the occipital lobes may have an earlier age of onset or a more aggressive disease course. We also assessed cortical thickness and administered detailed neuropsychological tests to understand the clinical implications of Aβ accumulation in the occipital lobes.

## 2. Materials and Methods

### 2.1. Participants

A total of 520 patients were clinically diagnosed as probable AD or amnestic mild cognitive impairment (aMCI) after completion of 3T MRI and neuropsychological tests between May 2013 and January 2016 at the Memory Disorder Clinic at Asan Medical Center, Seoul, Korea. The patients with probable AD dementia met criteria recommended by the National Institute on Aging–Alzheimer’s Association [1] and the patients with aMCI met the Petersen criteria for mild cognitive impairment [10]. The umbrella term, Alzheimer’s Continuum [2], was used to indicate both aMCI due to AD and AD dementia with amyloid PET positivity in this study [1]. Patients with diagnosis fulfilling posterior cortical atrophy were not included in the study [11]. We performed a [18F]-Florbetaben amyloid PET scan for a total of 121 subjects. A total of 75 subjects who completed the same diagnostic workups and fulfilled the diagnostic criteria of subjective memory impairment served as control subjects for cortical thickness analysis.

### 2.2. Ethical Approval

All procedures performed in studies involving human participants were in accordance with the ethical standards of the institutional research committee and complied with the principles of the 1964 Declaration of Helsinki and its later amendments or comparable ethical standards. Written informed consent was obtained from every subject prior to participation and also from the caregiver of the subject categorized as AD. The Institutional Review Board of Asan Medical Center approved the study protocol (2013-0847, 2014-0783, 2016-0588, 2016-0589, 2016-0590).

### 2.3. Demographics and Clinical Characteristics

We recorded the following information for each subject: age, sex, education level, history of hypertension (HT), diabetes mellitus (DM), hyperlipidemia, apolipoprotein E (APOE) state, age at onset, and disease duration. Age at onset was defined as the age when AD-related symptoms manifested, based on the report of the subject or an informant on the subject’s medical records. Disease duration was defined as the time from symptom onset to the time of [18F]-Florbetaben PET scan. Scores for the Korean version of the Mini-Mental State Examination (K-MMSE), Clinical Dementia Rating (CDR), CDR–sum of boxes (CDR–SB), and Global Deterioration Scale (GDS) were compared between the OCC+ and OCC− groups.

### 2.4. MRI Acquisition and Cortical Thickness Analysis 

All the participants underwent three dimensional (3D) T1-weighted MRI scans. T1-weighted volumetric MRI scans were acquired with a 3D gradient-echo T1-weighted sequence using a 3 Tesla MRI scanner (Achieva; Philips Medical Systems, Best, The Netherlands; echo time, 3.1 ms; repetition time, 6.8 ms; slice thickness, 1 mm with no gap; field of view, 270 mm; matrix size, 244 × 227 pixels). Images were processed using a standard Montreal Neurological Institute anatomic pipeline (ver. 1.1.9.; http://wiki.bic.mni.mcgill.ca/index.php/CIVET). The cortical thickness was measured as described previously [12]. Briefly, regional differences in cortical thickness of each group are shown at a false discovery rate corrected *p* < 0.05 with age, sex, education, and intracranial volume serving as covariates.

### 2.5. [18F]-Florbetaben Amyloid PET Acquisition and Analysis

The PET data were acquired using GE Discovery 690/710/690 Elite PET-CT scanners (GE Healthcare, Milwaukee, WI, USA) with profiles as described below (47 axial image planes; slice thickness, 3.27 mm; matrix size, 128 × 128; and transaxial field of view, 25 cm). A single dose of 300 MBq ± 20% [18F]-Florbetaben [13] was intravenously administered in a maximum volume of 10 mL. PET images were acquired from 90 to 110 min after injection with a three-dimensional (3D) list-mode acquisition setting on the scanners. The 5 min × 4 frames of PET scans were reviewed for any motion artifacts and motion-free frames were selected and summed. Finally, 20 min static PET images were used to obtain standard uptake values (SUV) and for further analysis.

### 2.6. [18F]-Florbetaben Amyloid PET Visual Assessment

We performed a visual rating of the [18F]-Florbetaben amyloid PET scans to determine whether each was global amyloid-positive or global amyloid-negative. Experts at the department of nuclear medicine (J.S.K. and M.O.), who were blinded to the diagnosis of each participant, assessed the PET images according to predefined Regional Cortical Tracer Binding (RCTB) and Brain Amyloid-beta Plaque Load (BAPL) scoring systems [4]. After this visual rating of global Aβ accumulation, we divided the global Aβ positive subjects into occipital Aβ positive (OCC+) and occipital Aβ negative (OCC−) groups, based on visual assessment of axial, coronal, and sagittal amyloid PET scans by two readers (J.H. and J.E.K.) blinded to the patients’ diagnoses. The occipital area used for visual assessment was defined medially by the parieto-occipital sulcus and laterally by an artificial line connecting the parieto-occipital sulcus and preoccipital notch. Cohen’s κ coefficient for inter-rater agreement over the division of subjects with amyloid imaging positivity into OCC+ and OCC− groups was calculated to be 0.70. In cases where there was any disagreement, the two readers reached a consensus after discussion.

### 2.7. Quantitative [18F]-Florbetaben Amyloid PET Analysis

Quantitative amyloid PET image analysis was conducted for all patients except for one, due to acquisition error of 3D-T1 MRI. FreeSurfer 5.1 was used for the generation of automatic volumes of interest (VOIs) for the quantitative PET analysis. First, the PET image of each patient was co-registered to the corresponding 3D-T1 MR image using a rigid-body transform-based registration method using Statistical Parametric Mapping 8 (SPM8, http://www.fil.ion.ucl.ac.uk/spm/software/spm8/). After the automated segmentation of structures, a correction was made when necessary, in accordance with the FreeSurfer manual (http://surfer.nmr.mgh.harvard.edu/fswiki/). Partial volume correction was not performed. The global SUV ratio (SUVR) was calculated for a merged group of lobar VOIs in AD signature areas (occipital, parietal, temporal, frontal, medial frontal, central, and posterior cingulate cortices, precuneus, and hippocampus/amygdala) using the cerebellar cortex as a reference [14,15].

### 2.8. Neuropsychological Test

All patients underwent neuropsychological tests using the Seoul Neuropsychological Screening Battery [16]. This included the following cognitive domain-specific functions: digit span forward and backward, for attention; the Korean version of the Boston Naming Test (K-BNT [16]), for language; the Rey–Osterrieth Complex Figure Test (RCFT) copy test, for visuospatial function; the Seoul Verbal Learning Test (SVLT) immediate recall, delayed recall task, and a recognition task, for verbal memory; RCFT immediate recall, delayed recall task, and a recognition task, for assessing visual memory function; and the phonemic and semantic Controlled Oral Word Association Test (COWAT [17]) and a Stroop Test (color reading), for frontal/executive function [18].

### 2.9. Visuoperceptual Function Tests

We made a composite test battery for the assessment of detailed visuoperceptual functions. This comprised tests for shape perception, angle perception, color perception, face perception, letter perception, visual agnosia, color agnosia, prosopagnosia, and reading. It included the Extended Complex Figure Test (ECFT) [17] for shape perception, a short version of the Benton Judgment Of Line Orientation test (JOLO) [18] for angle perception, a matching test of ten color plates for color perception, a facial discrimination subtest of the Korean version of the Florida Affect Battery (K-FAB) [19] for facial perception, the naming of ten color plates for color agnosia, and the Korean version of the naming of famous faces to assess prosopagnosia [20].

### 2.10. Statistical Analysis

We compared patients’ demographics and detailed neuropsychological test results between the OCC+ and OCC− groups. A simple κ statistic was used to assess the inter-reader agreement. The SUVR values in the ROIs in the brain were compared to see whether the SUVR in the occipital cortices was lower than that in the other regions using a repeated-measures ANOVA with regional SUVR as a within-subjects factor. The SUVRs in each brain ROI were compared between the OCC+ and OCC− groups using a linear regression model with age, sex, education, and global SUVR as covariates. For comparison between the groups, we used independent Student t-tests for continuous variables (age, education, age at onset, disease duration, K-MMSE score, and geriatric depression scale score) and χ2 tests for categorical variables (sex, diagnosis, APOE status, HT, DM, hyperlipidemia, and CDR). We applied linear regression analysis to each subscore of the neuropsychological tests with the disease state and global SUVR as covariates. A z-score adjusted for age and education was used for statistical analysis of the neuropsychological tests. We used SPSS version 21.0 (IBM Corp., Armonk, NY, USA) for the statistical analyses. Statistical significance was set as *p* < 0.05.

## 3. Results

### 3.1. Differences in Clinical Features between OCC+ and OCC- Groups

**Figure 1:**
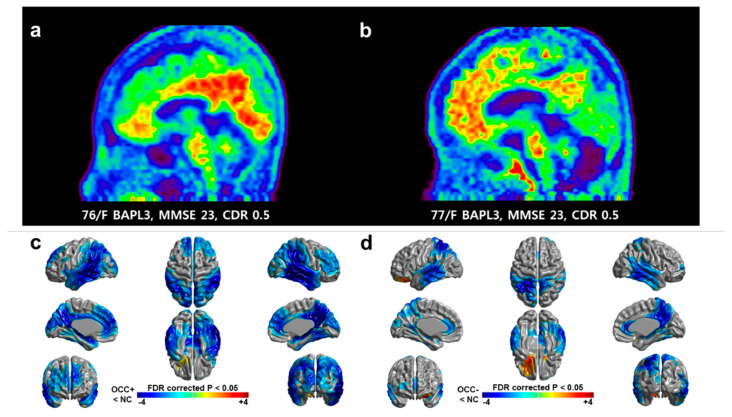
Representative [18F]-Florbetaben PET images and regional cortical thinning compared between the occipital Aβ positive group (OCC+) and occipital Aβ negative group (OCC−) groups. (**a**) [18F]-Florbetaben amyloid PET image of an occipital amyloid-beta (Aβ) positive patient. (**b**) [18F]-Florbetaben amyloid PET image of an occipital Aβ negative patient. (**c**,**d**) Regional differences in cortical thickness between each group and control are shown. Maps at a false discovery rate corrected *p* < 0.05 are shown with age, sex, education, and intracranial volume serving as covariates. The color scale bar represents the z-score.

Of the 121 patients, 74 (74/121, 63.6%) showed increased global amyloid uptake on the [18F]-Florbetaben PET scans. Out of these 74 patients, 41 (41/74, 55.4%) showed increased amyloid uptake in the occipital lobes, and 33 patients (33/74, 44.6%) did not (Figure 1a,b). The OCC+ group was younger (mean age = 68.3 ± 10.8 years) than the OCC− group (74.5 ± 6.5 years, *p* = 0.003), and the OCC+ group had an earlier age at onset (65.5 ± 11.1 years vs. 72.1 ± 7.1 years, *p* = 0.003), with an age range that was relatively compatible with early onset AD (EOAD; Table 1). The OCC+ group had higher GDS scores, a tendency to have more subjects with CDR 1, and higher CDR-SB scores, even though both groups had a similar disease duration (Table 1).

### 3.2. Differences in Neuroimaging Findings between OCC+ and OCC− Groups

Intriguingly, the quantitative measurement of [18F]-Florbetaben PET revealed that SUVR in the occipital lobes was higher than the SUVR in several AD signature areas, including the hippocampus/amygdala and temporal lobes. The SUVR in the occipital lobes was higher than the SUVR in primary cortices and was comparable with the value in frontal lobes in the OCC+ group (Table 2, upper table). As expected, that the OCC+ group had greater amyloid deposition in the bilateral occipital lobes than the OCC− group. Beyond the occipital area, the OCC+ group showed a higher SUVR globally, as well as regionally in the parietal, temporal, and precuneus areas (Table 2, lower table). The false discovery rate adjustment of *p*-values did not alter the statistical significance of the dataset. When compared to normal elderly subjects, the OCC+ group showed greater cortical thinning in the occipital area, as well as in the lateral parietal, lateral temporal, and precuneus regions, than the OCC− group (Figure 1c,d). Overall, the OCC+ group showed a substantial amount of amyloid uptake in the occipital lobes and greater regional and global amyloid uptakes with cortical thinning in relevant areas.

### 3.3. Differences in Neuropsychological Tests between OCC+ and OCC− Groups

The detailed neuropsychological tests revealed that the OCC+ group had a lower performance on RCFT copy, immediate recall, delayed recall, and recognition tests than the OCC− group. These findings remained significant even after treating disease state, or disease state and global SUVR, as covariates (Table 3). The additional visuoperceptual function tests revealed no statistically significant differences between the OCC+ and OCC− groups (Table 4). The ECFT score to assess visual perception was slightly lower in the OCC+ group than in the OCC− group when age, sex, education, disease state, and global SUVR served as covariates (*p* = 0.021).

## 4. Discussion

This study resulted in three main findings. First, we demonstrated that there was a substantial amount of amyloid deposition in the occipital lobes in Alzheimer’s Continuum patients at levels at least as high as or higher than in other AD signature regions. Second, the OCC+ group had a younger age at onset than the OCC− group, with the age range comparable to that for the onset of EOAD. Third, the OCC+ group had greater cortical thinning in the occipital lobes and adjacent areas, with greater impairment in visuospatial functions than the OCC− group.

In previous studies, considerable Aβ deposition was observed in the occipital lobes in patients with AD, although it was not noted or appreciated [4,5,6,7]. In the present study, we also observed significant deposition of Aβ in the occipital lobes, which was higher than medial temporal lobes, hippocampus/amygdala, and frontal lobes. This is probably due to the fact that the occipital cortices include not only primary visual cortices but also association cortices. The SUVRs in the primary motor and sensory cortices were lower than those in the occipital lobes. Based on the difference in clinical and neuroimaging characteristics between the OCC+ group and the OCC- group, we interpreted that the increased uptake in the occipital lobes has its own clinical implications. Many studies have reported that EOAD patients exhibited various characteristics in neuroimaging studies, such as atrophy in symptom-related areas [21], and greater global or parieto-occipital accumulation of Aβ [19]. In our previous study, we found that patients with greater atrophy in the parieto-occipital area were younger, had a younger age at onset, and had more Aβ accumulation in diffuse areas [12], findings that were comparable with those of early onset occipital Aβ accumulation in the present study. As EOAD is thought to have a more aggressive course than late onset AD (LOAD) [22], subjects with OCC+ in this study might have earlier onset of symptoms and a tendency to have worse global cognitive scores for a given disease duration.

When compared to normal elderly subjects, greater cortical thinning in the lateral temporal, lateral and medial parietal, and medial frontal cortices was noted in the OCC+ group than the OCC− group. This finding is comparable with previous studies that showed a difference in cortical thickness between patients with EOAD and LOAD [19]. In the present study, the visuospatial and visual memory functions differed significantly between the OCC+ and OCC− groups, which was compatible with corresponding cortical atrophy. A similar finding was observed in a previous study, in which occipital Aβ accumulation correlated with visuospatial dysfunction, although the visuoperceptual function was not evaluated in that study [23]. The visuoperceptual test results in the present study revealed no significant difference between the OCC+ and OCC− groups, even though the OCC+ group had greater Aβ accumulation in the occipital lobes and adjacent areas. These findings suggest that additional brain pathology, such as tau accumulation, would be required for the impairment of visuoperceptual function in Alzheimer’s Continuum [23].

Patients with PCA was not included in the study. Previous case reports have suggested higher occipital amyloid-PET uptakes in patients with PCA compared with typical AD [24,25,26]. However, other studies with larger numbers of subjects or longitudinal follow-up showed that the accumulation of Aβ in AD brain was widespread across association cortices, relatively non-specific to the presenting clinical features of AD, which correlated with tau accumulation [27,28,29]. Although differences may exist across studies, the existence of PCA needs to be considered in the case of increased amyloid uptake in posterior cortices of AD patients.

This study had several strengths, including thorough clinical investigation with visuoperceptual function tests and both qualitative and quantitative imaging analysis of amyloid on PET scans to assess occipital Aβ positivity. However, there were limitations. First, this was a cross-sectional study, so we could not observe the progression of clinical and image findings in the subjects with or without the accumulation of occipital Aβ. A longitudinal follow-up study would provide additional information regarding the changes in the occipital lobes and additional clinical implications. Second, we did not perform partial volume correction in this study, which may have affected the results. The small sample size was another limitation. Additional studies with larger numbers of participants are needed to confirm these results.

## 5. Conclusions

This study showed that AD and aMCI patients with occipital Aβ accumulation exhibited features noted in early onset AD; compared with patients without occipital Aβ accumulation, they had a younger age at onset and worse global cognitive score. These patients also had more impairment in the visuospatial and visual memory domains, reflecting cortical atrophy in relevant brain areas. Taken together, Aβ accumulation in the occipital lobes in amyloid PET scans needs to be recognized as being a potential biomarker of early onset AD dementia and relevant cognitive impairment.

## Figures and Tables

**Table 1:** Demographics and clinical characteristics.

	OCC+ (*n* = 41)	OCC− (*n* = 33)	*p*
Age (year)	68.32 ± 10.78	74.52 ± 6.52	0.003
Gender (Female)	31 (75.6%)	20 (60.6%)	0.166
Education (year)	10.37 ± 4.10	8.76 ± 4.91	0.129
Age at Onset (year)	65.49 ± 11.11	72.06 ± 7.09	0.003
Disease Duration (year)	3.22 ± 2.17	2.80 ± 1.90	0.389
HT	16 (39.0%)	23 (69.7%)	0.009
DM	3 (7.3%)	9 (27.3%)	0.021
Hyperlipidemia	17 (41.5%)	13 (39.4%)	0.857
Disease State			0.388
Alzheimer’s disease	24 (58.5%)	16 (48.5%)	
Amnestic MCI	17 (41.5%)	17 (51.5%)	
APOE E4 *			0.100
E4 carrier	16 (45.7%)	18 (66.7%)	
E4 non-carrier	19 (54.3%)	9 (33.3%)	
MMSE	20.46 ± 4.60	21.30 ± 4.64	0.440
CDR			0.070
0.5	20 (48.8%)	23 (69.7%)	
1	21 (51.2%)	10 (30.3%)	
CDR-SB	3.94 ± 1.88	3.18 ± 1.90	0.091
GDS	4.10 ± 0.80	3.67 ± 0.74	0.020
GDepS	13.97 ± 8.19	12.28 ± 7.41	0.369
Regional SUVR			
Occipital, Left	1.73 ± 0.20	1.49 ± 0.13	<0.001
Occipital, Right	1.75 ± 0.19	1.49 ± 0.15	<0.001
Global SUVR	1.73 ± 0.20	1.61 ± 0.16	0.005

OCC+, occipital Aβ positive group; OCC−, occipital Aβ negative group; HT, hypertension; DM, diabetes mellitus; MCI, mild cognitive impairment; APOE, apolipoprotein E; MMSE, Mini-Mental State Examination; CDR, Clinical Dementia Rating scale; CDR-SB, Clinical Dementia Rating scale—Sum of Boxes; GDS, Global Deterioration Scale; GDepS, Geriatric Depression Scale. The data are shown as mean ± SD or n (%). *APOE genotyping was performed in 62 patients.

**Table 2:** Quantitative [18F]-Florbetaben amyloid PET analysis in the occipital lobes.

**Aβ Accumulation in the Occipital Lobes Compared to Other Brain Regions**						
	**OCC+ (*n* = 41)**		**OCC− (*n* = 32)**		**Total (*n* = 73)**	
	**Mean ± SD**	***p***	**Mean ± SD**	***p***	**Mean ± SD**	***p***
Occipital	1.74 ± 0.19		1.49 ± 0.13		1.63 ± 0.21	
Parietal	1.85 ± 0.23	<0.001	1.70 ± 0.16	<0.001	1.79 ± 0.21	<0.001
Temporal	1.61 ± 0.20	<0.001	1.48 ± 0.15	0.639	1.55 ± 0.19	<0.001
Frontal	1.76 ± 0.22	0.535	1.66 ± 0.21	<0.001	1.71 ± 0.22	<0.001
Medial frontal	1.78 ± 0.22	0.187	1.68 ± 0.21	<0.001	1.73 ± 0.22	<0.001
PCC	1.98 ± 0.26	<0.001	1.87 ± 0.25	<0.001	1.93 ± 0.26	<0.001
Precuneus	2.03 ± 0.26	<0.001	1.89 ± 0.22	<0.001	1.97 ± 0.25	<0.001
Central	1.56 ± 0.18	<0.001	1.46 ± 0.13	0.052	1.52 ± 0.17	<0.001
Hippocampus and amygdala	1.23 ± 0.11	<0.001	1.21 ± 0.08	<0.001	1.22 ± 0.10	<0.001
**Aβ Accumulation between the OCC+ and OCC− Group**						
	**OCC+ (*n* = 41)**		**OCC− (*n* = 32)**		***p***	Adjusted *p* *
Occipital, Left	1.73 ± 0.20		1.49 ± 0.13		<0.001	<0.001
Occipital, Right	1.75 ± 0.19		1.49 ± 0.15		<0.001	<0.001
Parietal, Left	1.84 ± 0.24		1.71 ± 0.16		0.006	0.872
Parietal, Right	1.85 ± 0.22		1.70 ± 0.18		0.002	0.286
Temporal, Left	1.60 ± 0.21		1.49 ± 0.16		0.018	0.319
Temporal, Right	1.61 ± 0.19		1.48 ± 0.16		0.002	0.220
Frontal, Left	1.75 ± 0.23		1.66 ± 0.20		0.078	0.010
Frontal, Right	1.76 ± 0.21		1.65 ± 0.23		0.038	0.195
Medial frontal, Left	1.77 ± 0.23		1.68 ± 0.21		0.094	0.010
Medial frontal, Right	1.78 ± 0.22		1.67 ± 0.23		0.043	0.081
PCC, Left	1.97 ± 0.26		1.87 ± 0.27		0.099	0.616
PCC, Right	1.99 ± 0.26		1.88 ± 0.24		0.073	0.379
Precuneus, Left	2.04 ± 0.25		1.88 ± 0.25		0.011	0.417
Precuneus, Right	2.02 ± 0.28		1.89 ± 0.21		0.039	0.799
Central, Left	1.56 ± 0.19		1.46 ± 0.13		0.011	0.570
Central, Right	1.57 ± 0.18		1.46 ± 0.15		0.005	0.275
Hippocampus and amygdala, Left	1.23 ± 0.11		1.22 ± 0.08		0.457	0.258
Hippocampus and amygdala, Right	1.24 ± 0.10		1.21 ± 0.09		0.192	0.427

OCC+, occipital Aβ positive group; OCC− occipital Aβ negative group; PCC = posterior cingulate cortex. * Analysis adjusted for age, sex, education, and global SUVR.

**Table 3:** Neuropsychological test results.

	OCC+ (*n* = 41)	OCC− (*n* = 33)	*p*	Adjusted *p* *	Adjusted *p* ^†^
Digit Span Forward	−0.01 ± 1.04	−0.07 ± 0.98	0.795	0.681	0.843
Digit Span Backward	−0.57 ± 1.42	−0.42 ± 1.03	0.626	0.797	0.835
K-BNT	−1.71 ± 1.72	−1.70 ± 1.97	0.979	0.780	0.613
RCFT Copy Score	−4.20 ± 5.19	−1.04 ± 2.30	0.001	0.002	0.010
SVLT Immediate Recall	−1.62 ± 1.34	−1.53 ± 1.32	0.759	0.985	0.909
SVLT Delayed Recall	−2.46 ± 1.18	−1.99 ± 1.22	0.097	0.138	0.129
SVLT Recognition	−2.12 ± 1.78	−1.85 ± 1.20	0.432	0.586	0.407
RCFT Immediate Recall	−1.98 ± 0.82	−1.43 ± 0.80	0.005	0.007	0.018
RCFT Delayed Recall	−2.21 ± 0.85	−1.65 ± 0.83	0.006	0.009	0.014
RCFT Recognition	−2.77 ± 2.39	−1.38 ± 1.37	0.004	0.005	0.014
COWAT Animal	−1.53 ± 1.13	−1.14 ± 1.17	0.158	0.253	0.359
COWAT Supermarket	−1.15 ± 1.02	−0.94 ± 0.88	0.374	0.591	0.713
COWAT Phonemic	−0.86 ± 1.09	−0.73 ± 1.05	0.618	0.920	0.908
Stroop Color Reading	−2.15 ± 1.82	−1.60 ± 1.18	0.139	0.239	0.462

OCC+, occipital Aβ positive group; OCC−, occipital Aβ negative group; K-BNT, Korean version of the Boston Naming Test; RCFT, Rey–Osterrieth Complex Figure Test; SVLT, Seoul Verbal Learning Test; COWAT, Controlled Oral Word Association Test. The data are shown as z-scores, controlling for age and education. * Analysis adjusted for disease state. ^†^ Analysis adjusted for disease state and global SUVR.

**Table 4:** Visuoperceptual function test results.

	OCC+ (*n* = 12)	OCC− (*n* = 14)	*p*	Adjusted *p* ^a^	Adjusted *p* ^b^
ECFT	6.25 ± 1.71	7.57 ± 2.34	0.119	0.140	0.021
JOLO (Short Form)	6.50 ± 3.68	7.89 ± 3.98	0.377	0.397	0.131
K-FAB	17.50 ± 2.88	17.14 ± 3.39	0.777	0.867	0.082
Color Matching	9.75 ± 0.87	10.00 ± 0.00	0.339	0.519	0.563
Letter Matching	9.67 ± 0.89	9.79 ± 0.58	0.685	0.899	0.463
K-Famous Naming	20.33 ± 5.19	17.39 ± 7.18	0.185	0.408	0.425
Color Naming	9.36 ± 1.21	8.29 ± 2.46	0.167	0.212	0.658
Color Knowledge	18.46 ± 1.97	17.71 ± 2.05	0.281	0.601	0.823
Letter Reading	9.75 ± 0.87	9.86 ± 0.36	0.676	0.882	0.452
Word Reading	22.75 ± 2.34	23.21 ± 2.15	0.603	0.778	0.845

OCC+, occipital Aβ positive group; OCC−, occipital Aβ negative group; ECFT, Extended Complex Figure Test; JOLO, the Benton Judgment of Line Orientation test; K-FAB, Korean version of the Florida Affect Battery; K-Famous naming, Korean version of the naming of famous faces test. The data are shown as mean ± SD. ^a^ Analysis adjusted for age, sex, education, and disease state. ^b^ Analysis adjusted for age, sex, education, disease state, and global SUVR.

## Data Availability

Data will be available upon request.
